# Copper(II)-Assisted Degradation of Pheophytin *a* by Reactive Oxygen Species

**DOI:** 10.3390/ijms25031831

**Published:** 2024-02-02

**Authors:** Łukasz Orzeł, Agnieszka Drzewiecka-Matuszek, Dorota Rutkowska-Zbik, Aneta Krasowska, Leszek Fiedor, Rudi van Eldik, Grażyna Stochel

**Affiliations:** 1 Faculty of Chemistry, Jagiellonian University, 30-387 Cracow, Poland; aneta.krasowska@uj.edu.pl (A.K.); rudi.vaneldik@fau.de (R.v.E.); stochel@chemia.uj.edu.pl (G.S.); 2 Jerzy Haber Institute of Catalysis and Surface Chemistry, Polish Academy of Sciences, 30-239 Cracow, Poland; agnieszka.drzewiecka-matuszek@ikifp.edu.pl (A.D.-M.); dorota.rutkowska-zbik@ikifp.edu.pl (D.R.-Z.); 3 Faculty of Biochemistry, Biophysics and Biotechnology, Jagiellonian University, 30-387 Cracow, Poland; leszek.fiedor@uj.edu.pl; 4 Department of Inorganic and Coordination Chemistry, Nicolaus Copernicus University, 87-100 Torun, Poland; 5 Department of Chemistry add Pharmacy, University of Erlangen-Nuremberg, 91054 Erlangen, Germany

**Keywords:** pheophytin *a*, copper(II), reactive oxygen species, linear tetrapyrrole formation

## Abstract

The central ion Mg^2+^ is responsible for the differences between chlorophyll a and its free base in their reactivity toward metal ions and thus their resistance to oxidation. We present here the results of spectroscopic (electronic absorption and emission, circular dichroism, and electron paramagnetic resonance), spectroelectrochemical, and computational (based on density functional theory) investigations into the mechanism of pheophytin, a degradation that occurs in the presence of Cu ions and O_2_. The processes leading to the formation of the linear form of tetrapyrrole are very complex and involve the weakening of the methine bridge due to an electron withdrawal by Cu(II) and the activation of O_2_, which provides protection to the free ends of the opening macrocycle. These mechanistic insights are related to the naturally occurring damage to the photosynthetic apparatus of plants growing on metal-contaminated soils.

## 1. Introduction

The electronic and coordinative properties of chlorophylls (Chls) and bacteriochlorophylls (BChls) are strongly influenced by the character of species bound in their central cavity, which, in most cases, is occupied by the Mg^2+^ ion [1,2,3]. Due to the special non-coordinative type of binding, it plays a key role in taming the intramolecular intersystem crossing in Chls [4,5]. Importantly, the removal of the central metal ion affects the redox properties of Chls and thus its free base, pheophytin a (Pheo*a*), functions exclusively as the primary electron acceptor in the photosystem II reaction center. On the other hand, the replacement of the central Mg^2+^, e.g., in chlorophyll *a* (Chl*a*), with other divalent metal ions impairs the photochemical properties of the pigment and most often leads to the inhibition of photosynthesis. Such a spontaneous substitution is observed in plants growing in soils contaminated with Cu, Cd, or Hg compounds, and causes irreversible disruption to the electron transport chain within the thylakoid membranes [6,7,8]. Therefore, sophisticated protective mechanisms of natural photosystems, supported by some endogenous ligands, shield pigments from the redox activity of metallic impurities taken up from the environment [9]. On the other hand, the metallo-derivatives of chlorophylls and bacteriochlorophylls are desirable for their potential biomedical [10,11,12] or photocatalytic [13,14,15] applications.

We have shown that the routes of Chl*a* reactions with redox-active metal complexes (Cu(II) in particular) strongly depend on the medium. Besides transmetalation, i.e., the substitution of the central Mg ion and the metalation of Pheo*a*, two fundamentally distinct redox pathways can be followed [16]. The redox cycling is characteristic of Chl*a*, which is alternately oxidized to a cation radical and reduced back in a reaction involving mixed Cu(I) and Cu(II) species. An important role in these reactions is played by reactive oxygen species (ROS), namely superoxide and peroxide, whose generation and scavenging are strictly correlated with the Chl*a* redox cycling and Cu(II)/Cu(I) speciation equilibria [17].

In the case of Pheo*a*, similar redox cycling can only be observed under very specific conditions as a reaction parallel to the dominant degradation of the pigment. In a previous article we identified the boundary conditions for Pheo*a* degradation in the presence of various Cu(II) complexes [16]. Our spectroscopic and electrochemical studies revealed that the degradation of Pheo*a* in weakly coordinating organic solvents prevails in large excesses of Cu(II) salts with weakly coordinating counterions in equilibrium with air. In this study we focus on the mechanism of this complex process.

## 2. Results and Discussion

**Figure 1:**
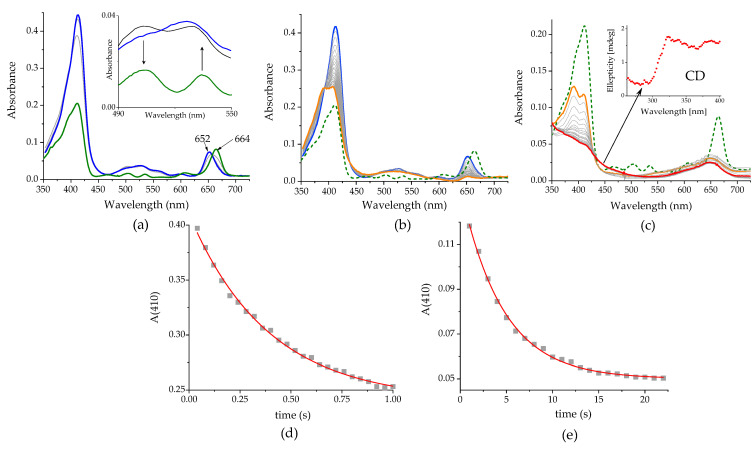
Changes of the UV-Vis spectra accompanying the reaction between Pheo*a* and CuTf_2_ (1:2000) in MeCN. (**a**) 0–20 ms, inset: zoom at 490–550 nm; (**b**) 0–1 s; (**c**) 1–25 s, inset: the circular dichroism (CD) spectrum of the reaction mixture recorded 30 s after the mixing of the reactants (the arrow indicates corresponding CD and UV-Vis spectra). The colored lines represent the spectrum recorded before (green), 25 ms (blue), 1 s (orange) and 25 s (red) after mixing the reagents. The kinetic traces recorded at 410 nm for the reaction steps shown in (**b**,**c**) are presented in (**d**,**e**), respectively.

***The effect of* Cu(II).** A series of spectroscopic and computational techniques was applied in order to follow the course of the reaction of Pheo*a* with CuTf_2_, which constitutes a convenient model system [16]. The time resolution of the stopped-flow spectrophotometer (in a rapid-scan mode) allowed us to resolve three reaction steps. The first one (step 1) was the quickest and, under set conditions ([CuTf_2_]/[Pheo*a*] = 2000, 25 °C), was completed while the reactants were being mixed. The changes occurring during this step concern all the absorption bands in the visible range. While the intensity of the Soret band increases very significantly, the bands in the 450–500 range change their relative intensity, and the Q_Y_ band shifts hypsochromically by 12 nm (Figure 1a).

Step 2 of the reaction lasts about 1 s. During this time, one could observe a ~50% reduction in the intensity and clear separation of the two components of the Soret band, flattening of the weak central bands, and almost a complete loss of the Q_Y_ band (Figure 1b). The overall reaction is complete in another approx. 20 s. In the slowest step, step 3, the Soret band decays to its residual form. As a result, the spectrum of the product consists of two weak but very broad bands stretched over almost the entire visible range of the spectrum (Figure 1c).

The multi-step reaction of Pheo*a* with CuTf_2_ is reflected in quite characteristic changes in the absorption spectra, pointing to the differing nature of the changes occurring at each step. The blue shift of the Q_Y_ band to c. 652 nm observed in the initial, very fast step is indicative of the formation of the copper analogue of Chl*a* (Cu-Pheo*a*). However, the time scale seems to be too short for the complete metalation of Pheo*a*. Formation of Cu-Pheo*a* is also unlikely due to the presence of the bands in the 450–550 nm range that reflect symmetry, which can be achieved either by the coordination of the metal ion out of the plane, or by the protons remaining in the cavity of the macrocycle. Some similarities of these spectral shifts to those reported for the allomerization of Chls may indicate its occurrence in our system, as a reaction parallel to the degradation of the macrocyclic ring, pointing to the presence of O_2_^•−^ [18,19].

The considerable simplification of the absorption spectrum, primarily in its central part, observed only during step 2, results in the spectrum taking on features more typical of porphyrin than of chlorin. A change like this requires the two-electron oxidation of the macrocyclic ligand. The complete bleaching which occurs in step 3 is a clear indication of the definitive breakdown of the macrocyclic system. An open-chain species of tetrapyrrole, bilin, is the expected product, as suggested by earlier studies on the reactivity of photosynthetic pigments toward oxidizing agents [20,21].

The mechanism, proposed by us, of Pheo*a* breakdown leading to a linear tetrapyrrolic product is strongly supported by both the chirality and the shape of the circular dichroism spectrum of the reaction mixture recorded shortly after the mixing of the reactants (Figure 1c). The initial signals of the substrate (Pheo*a*) quickly disappear and the resulting spectrum shows two maxima, near 290 and 320 nm, respectively, which is very characteristic of the NCC-type (“nonfluorescent chlorophyll catabolite”, open chain) breakdown product of pheophorbide *a* [22]. We think that the chirality of the product and its characteristic CD spectrum are clear evidence for the specific site of the macrocycle opening.

**Figure 2:**
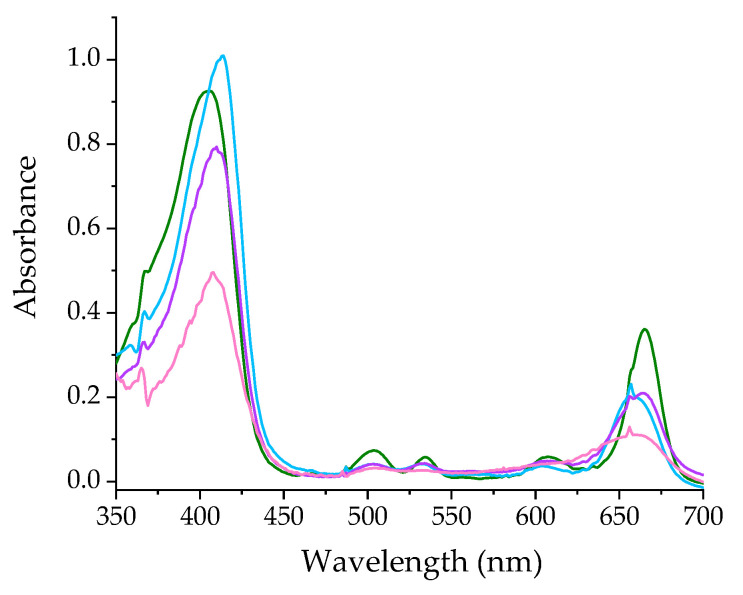
Electrochemically induced changes in the UV-Vis spectrum of Pheo*a* in MeCN. Applied voltage: 0.0 V (green line), +0.5 V (light blue line), +1.0 V (violet line) and +1.5 V (pink line).

To identify the oxidation step of the pigment, an experiment was performed in which a solution of Pheo*a* in MeCN was subjected to a voltage varying from 0 to +1.5 V in 0.5 V intervals. The absorption spectra recorded after equilibrium was established in the solution are shown in Figure 2.

With an initial increase in the voltage, a slight increase in the Soret band and a c. 40% decrease in the Q_Y_ band is observed. These changes, as well as the shift of the Q_Y_ band maximum toward shorter wavelengths, resemble those observed in step 1 of the reaction with Cu(II) (compare light blue line in Figure 2 and blue line in Figure 1a). Further raising the voltage to +1.0 and +1.5 V causes a gradual decrease in absorbance in all the pigment’s bands. The shape of the last one (pink line) features only the remnants of the Q_Y_ band, which is clearly broadened and shifted toward shorter wavelengths. All these features make this spectrum similar to that of the reaction product with CuTf_2_. However, the still relatively intense and narrow Soret band points to step 2 as being the one in which the electron transfer occurs. The less pronounced effects and inaccurate reproduction of the shape of the Soret band in the spectroelectrochemical experiment are probably due to both the low efficiency of the electrode reaction and the exclusion of changes resulting from metal coordination.

**Figure 3:**
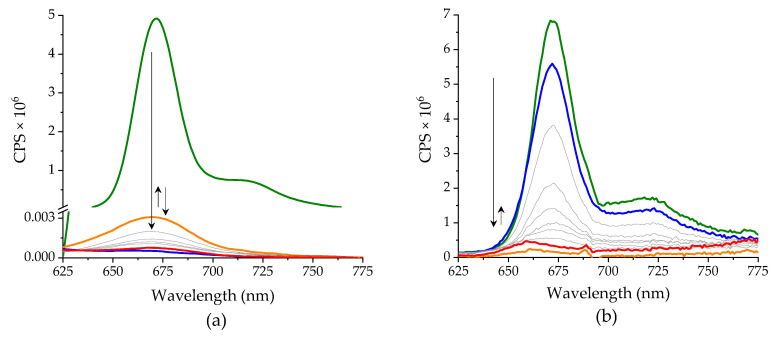
Changes in fluorescence emission of Pheo*a*: (**a**) accompanying the reaction with excess CuTf_2_ in MeCN and (**b**) induced electrochemically by subjecting the solution gradually (in +0.5 V increments) up to a voltage of +1.5 V. Colored lines refer to the spectra recorded: before (green), during (blue and orange, successively) and after the experiment (red). Arrows indicate changes in band intensity (sequentially from left to right).

Some additional information on the mechanism of the reaction of Pheo*a* with CuTf_2_ was provided by the recorded emission spectra (Figure 3a). A nearly complete decay of the fluorescence takes place, even during the mixing of the reagents (blue line). Surprisingly, in the following somewhat slower step the emission is slightly restored with the preservation of the initial band position (orange line). The non-fluorescent product is formed in the final step of the reaction (red line). In a separate experiment, the spectra of the pigment subjected to an increasing voltage were recorded (Figure 3b). Despite the significant differences in the dynamics and magnitude of the ongoing changes, the trend is essentially similar to that in the reaction with CuTf_2_. In both cases, an almost complete disappearance of the fluorescence of the pigment is observed, although much slower in the electrode reaction than in the presence of Cu(II) ions. Furthermore, the subsequent, very slight increase in emission intensity makes the two systems similar. Despite these similarities, the interpretation of the reaction with CuTf_2_ is not quite straightforward. The initial decay of the emission may also result from the oxidation of Pheo*a* to its cation radical, or from the quenching of the excited fluorophore within the outer-sphere complex. In turn, the subsequent temporary increase in intensity may be due to the reduction of coordinated Cu(II) to Cu(I). Nevertheless, the ultimate loss of emission ability is characteristic of the formation of the linear forms of tetrapyrrole [23,24].

**Figure 4:**
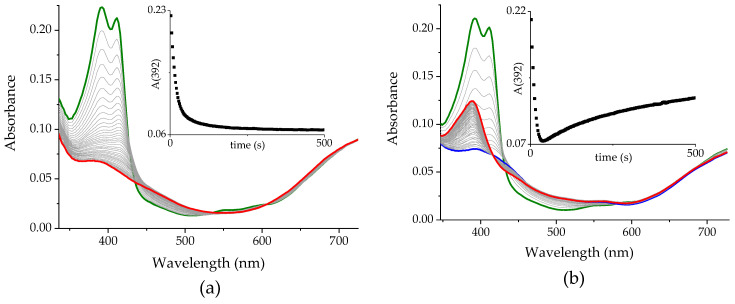
Changes in the UV-Vis spectra of Pheo*a* accompanying the reaction with CuTf_2_ in (**a**) O_2_-saturated and (**b**) deoxygenated MeCN solution. Insets: kinetic traces at 392 nm. T = 298 K. Colored lines refer to green—before mixing, blue—right after mixing, and red—last recorded spectrum.

***The effect of* O_2_**. The decomposition of the Pheo*a* macrocyclic system is due to the action of an oxidant, which in the system under investigation can be either the Cu(II) complex or some reactive oxygen species. To clarify this doubt, the effect of O_2_ on the reaction of Pheo*a* with CuTf_2_ in MeCN was tested. No changes were found during the long-term spectroscopic observations of the pigment in solutions with different O_2_ contents. Reactions with CuTf_2_ were carried out successively in equilibrium with air, as well as in deoxygenated and O_2_-saturated solutions. In the presence of dissolved O_2_, regardless of its concentration, the observed spectral changes have the same pattern (cf. Figure 1c and Figure 4a). The observed rate constant for the slower step of the reaction in the O_2_-saturated solution (k_obs_(O_2_) = (6.11 ± 0.05) × 10^−2^ s^−1^) is only slightly higher than for the reaction in equilibrium with air (k_obs_(air) = (5.99 ± 0.03) × 10^−2^ s^−1^). Changes found in the deoxygenated solution are of a slightly different nature (Figure 4b). The initial decay of all absorption bands is similar but noticeably faster than in the other systems (k_obs_(Ar) = (9.99 ± 0.16) × 10^−2^ s^−1^). This is followed by a much slower recovery of the band in the range typical of Soret (388 nm).

The rapid and almost complete bleaching observed in the first step indicates that the oxidative degradation of the macrocyclic ligand occurs via the reaction with the Cu(II) complex, and does not require the participation of O_2_. Restoration of the absorption band at 388 nm, typical of porphyrinoids, suggests that the macrocyclic system can be reconstituted from the linear species of tetrapyrrole. This is only possible if the terminal carbon atoms remain unrestricted. The well-known products of Chl*a* degradation that appear in its metabolic pathway, namely bilins, have two carbonyl groups formed by the attachment of two oxygen atoms [25]. In turn, anaerobic conditions ensure the possibility of the recombination of the radicals on the carbon atoms generated via homolytic bond cleavage. In addition, restoration of this bond can be sterically facilitated in the presence of a metal ion preferring planar or octahedral coordination, such as Cu(II).

**Figure 5:**
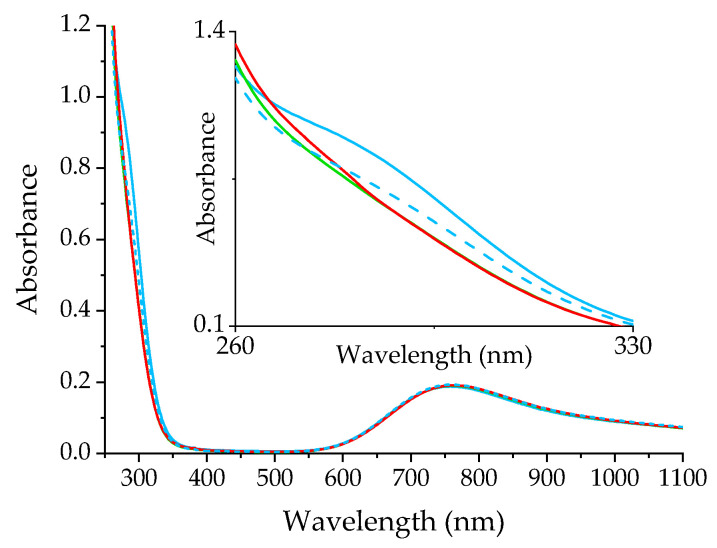
The effect of oxygen on the UV-Vis spectrum of CuTf_2_ in MeCN. The solution in equilibrium with air (blue solid line) was successively deoxygenated (green line), saturated with O_2_ (red line), and allowed to equilibrate with air again (blue dashed line).

Additional information on the role of O_2_ was gained from a brief study of its effect on Cu(II) speciation. The absorption spectra of CuTf_2_ were recorded sequentially in MeCN solution equilibrated with air, deoxygenated, saturated with O_2_, deoxygenated again, and left to re-establish equilibrium with air (Figure 5). While the change in O_2_ concentration has virtually no effect on the electronic transitions located on metal orbitals (>600 nm), some effect becomes apparent in the range of CT bands. The removal of O_2_ from the solution is accompanied by the disappearance of a low-intensity band at 280 nm, which is restored when the system reaches equilibrium with the air again (Figure 5).

The dependence of the appearance of this band on dioxygen concentration points to the interaction of the O_2_ with the metal ion. Consequently, a certain fraction of Cu(I) is expected to be present in the system, which is somehow confirmed by a relatively weakly intense band of d–d transitions. This is quite usual in solutions favoring Cu cluster formation [26,27]. Although this system is not particularly efficient in this regard, a small amount of Cu(I) can coexist and be sufficient to activate O_2_,

(1) $${Cu}^{I}+O_{2}\to {Cu}^{II}(O_{2}^{-})$$

which, in this way, becomes capable of blocking the intersection sites of the macrocycle. The smaller contribution of Cu^II^(O_2_^−^) in the O_2_-saturated solution than in equilibrium with air is probably mainly due to the almost complete conversion of Cu(I) to Cu(II). However, it is reasonable to assume that superoxide formation is favored in the presence of Pheo*a*, which increases the contribution of Cu(I) to the copper speciation equilibrium.

**Figure 6:**
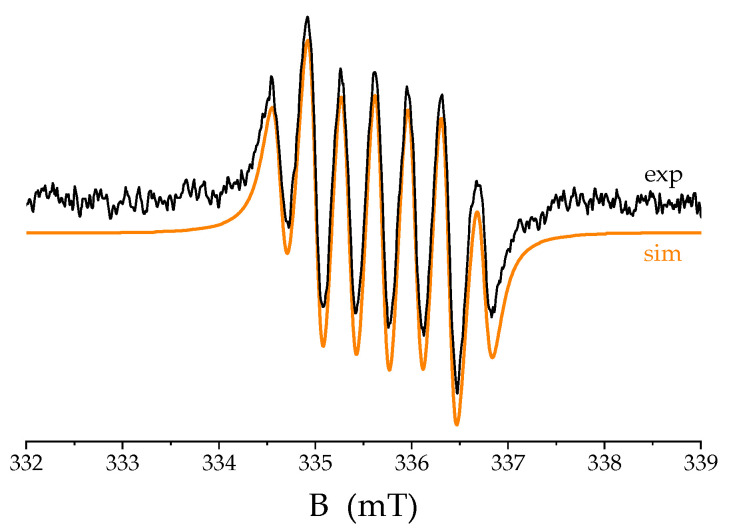
EPR spectrum of DMPO in air-equilibrated CuTf_2_ solution in MeCN. Black line—experimental trace, orange line—simulation.

We attempted to check the possibility of the activation of O_2_ and identify its reactive species in solution via EPR spectroscopy using DMPO as a spin trap. The recorded spectrum ruled out the existence of any free ROS in the absence of Pheo*a*. However, a few minutes after adding DMPO, a signal appeared with hyperfine structure parameters, namely aiso(N) = 0.69 mT, and aiso1(H) = aiso2(H) = 0.37 mT [1], indicating the formation of DMPOX (5,5-dimethyl-2-pyrrolidone-N-oxyl) (Figure 6), which is characteristic for the system containing the metal-bound oxygen radical [28,29,30]. This argues for the presence of Cu^II^(O_2_^−^) and, at the same time, for the occurrence of Cu(I) in the solution.

**Figure 7:**
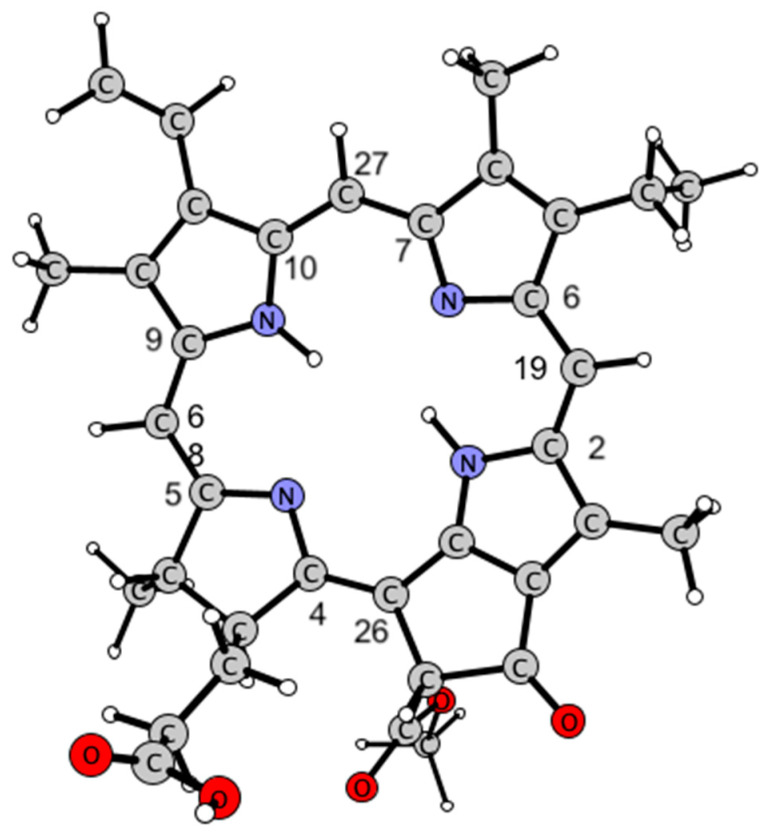
Structure of Pheo*a* and the atom numbering used in the text.

***Theoretical approach to the interactions in the Pheo*a*/copper/superoxide system.*** The DFT studies were performed to determine the effect of electron withdrawal on the stability of the macrocyclic system. The comparison of the bond lengths of the methine bridges in Pheo*a* (Table 1; see Figure 7 for the model used) and its two-electron oxidized form (Pheo*a*^2+^) indicate that while some of them are elongated (by 0.033 Å on average), the rest are somewhat shortened (by 0.016 Å on average), leading to the slight expansion of the macrocycle (all changes result in a 0.083 Å longer circuit).

**Figure 8:**
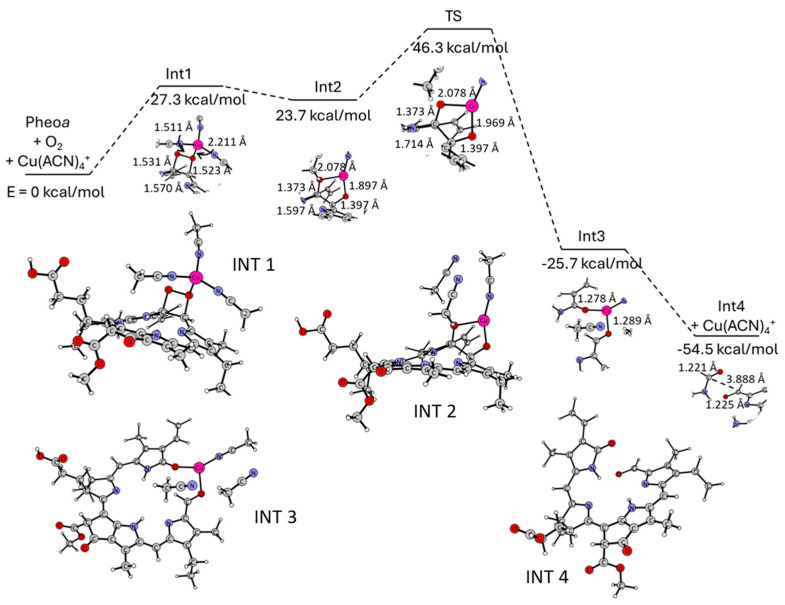
DFT:BP/def2-TZVP energy diagram for the proposed transformation of Pheo*a* into an open-chain species. Proposed intermediate states (INT 1–4) are shown below.

We attempted to gain a closer look at the subsequent changes in the reaction system The energy diagram of the reaction and the possible structures which can be formed during the interaction between Pheo*a*, solvated copper ion, and dioxygen are presented in Figure 8.

At first, the dioxygen can be bound to the methine bridge, forming a cyclic peroxide species (INT1, Figure 8) [31]. This leads to the methine C-C bond elongation from 1.392 Å to 1.570 Å. At the same time, the O-O bond is stretched from 1.22 Å (being its length in the isolated O_2_) to 1.511 Å. The C-C bridge can then be broken, together with the O-O bond of the dioxygen, which is accompanied by the insertion of the Cu ion in between two oxygen atoms, and its partial deligation (INT2, Figure 8). In the resulting intermediate, the methine C-C bond length is equal to 1.597 Å, and the Cu-O bonds to 2.078 and 1.897 Å. The C-O bonds are 1.373 Å and 1.397 Å long. Next, the methine bridge is broken and the distance between the two carbon atoms increases to 3.781 Å (INT3, Figure 8). In the transition state, the C-C bond length is equal to 1.714 Å, and the energy barrier for this step is equal to 22.6 kcal/mol (Figure 8). The copper ion plays the role of a pin holding together two oxygen atoms. The latter form bonds with the respective carbon atoms of 1.289 Å and 1.278 Å. Their length indicates that two carbonyl groups are formed. Finally, 1-formyl-19-oxobilane is formed (INT4, Figure 8). The macrocyclic ring is opened, and two oxygen atoms form two carbonyl groups of typical lengths (1.221 Å and 1.225 Å). Overall, this process is accompanied by a decrease in the total energy, showing that the resulting 1-formyl-19-oxobilane is more stable than Pheo*a*.

***The mechanism of Pheo*a *breakdown***. To gain more insight into the mechanism of the reaction of Pheo*a* with CuTf_2_ in MeCN, we performed kinetic investigations in air-equilibrated solution. Only two of the three recognized reaction steps were accessible, due to their time scale. Step 1 (Figure 1a) was completed while the reagents were being mixed in stopped-flow experiments. Thus, it proved to be too fast even for this technique.

**Figure 9:**
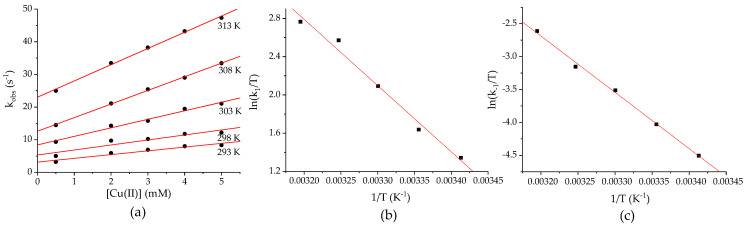
(**a**) The k_obs_ dependences on CuTf_2_ concentration for step 2 of the reaction with Pheo*a* (2.5 μM) in MeCN. The Eyring plots in (**b**,**c**) were constructed using k_1_ and k_2_ values, respectively. Kinetic data were determined in equilibrium with air at ambient pressure.

The spectroscopically recognized step 2 (cf. Figure 1b) is completed within a few seconds. The recorded time traces were satisfactorily fitted with mono-exponential functions. A large excess of CuTf_2_ over Pheo*a* was used to ensure pseudo-first-order conditions. The dependences of k_obs_ on CuTf_2_ concentration at 298–313 K are shown in Figure 9a.

The linear course of concentration dependence with a clear intercept indicates that this reaction step follows the rate law:

(2) $$k_{obs}=k_{1}\left[Cu\left(II\right)\right]+k_{2}$$

where k_1_ is the second-order rate constant for complex formation. The value of k_2_ is usually attributed to the back reaction, although, in this case, such an interpretation is not completely straightforward. The coexistence of Cu^II^(O_2_^−^) species with the predominant solvato complex of Cu(II) gives rise to the implementation of the competitive reaction pathways leading to the formation of the product (Cu-Pheo*a*), one of which (represented by k_1_) is dependent on, and the other (represented by k_2_) independent of, the metal concentration. The activation parameters were obtained according to the Eyring equation. The temperature dependences for the k_1_ and k_2_ values are shown in Figure 9b,c, respectively, whereas Table 2 presents the determined kinetic parameters. The small positive values of the activation entropy (ΔS^‡^) indicate a dissociative-interchange mechanism of both reactions. In addition, there is not much difference between the values of activation enthalpy for the [Cu(II)]-dependent and [Cu(II)]-independent reaction.

Kinetic studies of the subsequent step 3 were carried out following the decay of the Soret band. Kinetic traces of a bi-exponential nature were recorded, although no dependence of the rate of this step on Cu(II) concentration was found.

The pattern of spectroscopic changes along with the time scales of the respective steps allowed us to propose the following sequence of reactions.

The spectroscopic features observed in step 1 prompt us to assign them to Pheo*a* allomerization, which is presumably accompanied by the formation of an outer-sphere complex, within which charge relocation can occur:

(3) $$Pheoa+{Cu}^{2+}⇄\left\{Pheoa\cdot \cdot \cdot {Cu}^{2+}\right\}$$

(4) $$\left\{Pheoa\cdot \cdot \cdot {Cu}^{2+}\right\}\;\;⇄\;\left\{{Pheoa}^{•+}\;\cdot \cdot \cdot {Cu}^{+}\right\}$$

In the spectroscopically recognized step 2 (cf. Figure 1b), the reduced Cu center binds O_2_, while the outer-sphere adduct presumably transforms into an actual complex:

(5) $$\left\{{Pheoa}^{•+}\;\cdot \cdot \cdot {Cu}^{+}\right\}+O_{2}\to {\left[Pheoa-CuO_{2}\right]}^{2+}$$

The final step, step 3, must consist of all the complex electronic and structural transformations that lead to the final breakdown of the macrocyclic system. Since its rate does not depend on Cu(II) concentration (and also, essentially, on [O_2_]), all transformations must occur within the already formed Pheo*a*-Cu(O_2_) complex.

(6) $${\left[Pheoa-CuO_{2}\right]}^{2+}\to {\left[Pheoa-CuO_{2}\right]}^{{2+}^{\prime }}$$

Although our experimental results did not provide the exact specification of the intermediate species (represented by [Pheo*a*-CuO_2_]^2+^′) formed at this step of the reaction, particular clues were provided by the computational methods used. According to them, a species can appear in which the breaking C-C bond is “pinned” by the O-Cu-O system (compare Figure 9c). The terminal carbonyl groups thus formed eventually stabilize the product and the metal ion can be released.

(7) $${\left[Pheoa-CuO_{2}\right]}^{{2+}^{\prime }}\to Pheoa{\left(O\right)}_{2}+{Cu}^{2+}$$

In our earlier studies [16], we determined the boundary concentration ratios of Cu(II) complexes and Pheo*a* at which pigment degradation occurs, regardless of the presence of O_2_. In light of the current findings, it seems reasonable to expect that limited O_2_ access may allow the reconstitution of some form of macrocyclic system aided by coordination around the copper ion. This hypothesis requires further investigation.

## 3. Materials and Methods

**Pheophytin *a*.** The cyanobacterium *Arthrospira maxima* originating from the Culture Collection of Autotrophic Organisms in Trebon (Czech Republic) was used as a source of photosynthetic pigments employed in our studies. Chl*a* was extracted using Iriyama’s method [32] and subjected to two-stage purification. Column chromatography on DEAE sepharose (Sigma, Darmstadt, Germany) was employed in the first step [33]. In the second step, isocratic reversed-phase high-performance liquid chromatography with MeOH as the eluent was used. The HPLC setup (Varian, Palo Alto, CA, USA) was equipped with a ProStar 230 pump, an RP-C18 column (250 mm × 10 mm and a flow rate of 4 mL/min), and a TIDAS diode array detector (J&M, Frankfurt am Main, Germany) for the online monitoring of the absorption spectra. Pheophytin *a* was prepared using Chl*a* with a small amount of doubly distilled glacial acetic acid at room temperature for a short time, according to the procedure previously described [34]. The acid was removed in a stream of nitrogen. The product was dried under a vacuum and quickly purified using column chromatography on DEAE-Sepharose in acetone. The purity of Pheo*a* was confirmed using reversed-phase HPLC. The pigment was stored under argon at –20 °C. All experiments were performed in dimmed light with freshly prepared solutions.

**Solvents and Reagents.** Copper(II) trifluoromethanesulfonate (CuTf_2_) and acetonitrile (MeCN), both of analytical grade, were purchased from Sigma-Aldrich (Merck, Darmstadt, Germany). The gases Ar and O_2_ (5.0 grade) were obtained from Air Liquid.

**Spectroscopic and Kinetic Measurements.** A Lambda 35 and Lambda 950 (Perkin Elmer, Waltham, MA, USA) spectrophotometer, both equipped with a Peltier temperature controller PTP-6, were used both in the preparation of Pheo*a* solutions of a certain concentration and in testing the effect of O_2_ on Cu(II) speciation. Depending on the requirements, the experiment was carried out in a 1 cm quartz cuvette or in a screwed quartz cuvette with a silicone rubber septum (for anaerobic conditions).

The progress of the reaction was followed using an SX20 (Applied Photophysics, Surrey, UK) stopped-flow spectrophotometer equipped with a photodiode array detector. The rapid-scan technique was applied to record spectral changes in the UV-Vis range. To determine the reaction rates, single wavelength kinetic traces at 652 nm (step II) and 410 nm (step III) were collected. The temperature was controlled using a Labo Plus (Polyscience, Warrington, PA, USA) thermostatic bath. The oxygen-saturated and deoxygenated systems were obtained by purging the reagent solutions with O_2_ and A, respectively. The reactions were monitored using the rapid-scan technique. All data obtained in stopped-flow experiments were processed using the software Pro-Data SX v. 2.2.27.

The emission spectra were recorded on a Fluorolog 3 (Horiba—Jobin Yvon, Edison, NJ, USA) spectrofluorometer in 1 cm quartz cuvettes. The samples were excited at 410 nm. The spectra were recorded in the 600–800 nm range at equal time intervals and processed using the software OriginPro 2020 (Academic).

The spectroelectrochemical experiments were performed using an SP150 (BioLogic, Seyssinet-Pariset, France) potentiostat combined with a Lambda 265 (Perkin Elmer) spectrophotometer and Fluorolog-3, to track the effect of the potentials applied on the absorption and emission changes, respectively. A platinum grid was used as a working electrode, an Ag/AgCl cell as a reference electrode, and a thin platinum rod as an auxiliary electrode. The spectrum of c. 10 μM Pheo*a* in the presence of 0.1 M lithium perchlorate (used as a supporting electrolyte) was recorded and then the potential of +0.5 V was applied and maintained for 5 min., during which the spectra were collected at equal time intervals. This procedure was repeated successively with the potential increasing by 0.5 V up to +1.5 V.

The EPR experiment was performed on a Miniscope MS 400 (Magnettech, Berlin, Germany) spectrometer with DMPO as the spin-trapping agent (solution concentration 40 mg/mL). The instrumental parameters were as follows: X-band microwave frequency ~9.43 GHz; sweep time 120 ms; time constant 0.1 ms; modulation amplitude 0.05 mT; and microwave power 5 mW. Computer simulation of the experimental spectrum was performed with the software EPRsim32 [35].

The circular dichroism spectra were recorded on a J-815 spectropolarimeter (JASCO, Tokyo, Japan) in a 1 cm pathlength quartz cell, under ambient conditions, at a scan rate of 200 nm/min with an increment of 2 nm.

**DFT.** The computational studies were carried out using density functional theory (DFT), as implemented in Turbomole v7.0 [36]. The gradient-corrected Burke–Perdew (B–P) functional [37,38,39] was applied, with a def2-TZVP basis set [40] for all atoms. The resolution of identity (RI) algorithm [41,42] was applied for computing the electronic Coulomb interactions. The computations involved geometry optimization of the structures, further confirmed via vibrational analysis to ascertain whether the resulting structures were the minima on the potential energy surface. In each case, two multiplicities were considered. The electronic properties of the systems were studied with the aid of natural population analysis (NPA) [43].

## 4. Conclusions

The mechanisms of the reactions between Chls and transition metal ions have attracted interest because of both the applicability of porphyrinoid metallo-complexes [44,45] and their relevance to the damage of photosynthetic apparatus that occurs in nature [24,46]. Our model studies provide the basis for a better understanding of the role of the intracellular environment, such as functional side groups of amino acids in the Chl-binding proteins, in controlling the reactivity of the metal ions toward photosynthetic pigments, as well as in the design of novel functional bio-mimetic systems. The present results confirm the cooperativity of the O_2_ and Cu(II) ions in the oxidative breakdown of Pheo*a*. Three reaction steps distinguished in the spectroscopic studies indicate a stepwise oxidation of the macrocycle, in which Cu(II) presumably plays the role of the primary electron acceptor. Its reduction to Cu(I) allows the binding and transformation of O_2_ to a superoxide species, which, remaining in proximity to the macrocycle due to the binding to the central Cu ion, undergoes cleavage, along with the macrocycle cleavage at a specific methine bridge. Thus, a stable open-chain NCC-like product is formed, as evidenced by its circular dichroism features. In addition to the complex formation [9,47] and the redox cycling characteristic of Chl*a* [17], the mechanism revealed here represents another pathway of reactions between Chls and transition metal ions, an alternative to those traditionally considered. This may also partly account for the role of heavy metallic pollutants in damaging the photosynthetic apparatus in plants. On the other hand, the propensity of the Chl-free base to undergo such a reaction is yet another argument in the discussion of the role of the central Mg ion in Chls.

## Figures and Tables

**Table 1:** Selected bond lengths (in Å) of methine bridges in Pheo*a* and Pheo*a*^2+^. For atom notation see Figure 2.

Bond	Pheo*a*	Pheo*a*^2+^
C10–C27	1.392	1.375
C27–C7	1.407	1.431
C9–C68	1.402	1.410
C68–C5	1.397	1.385
C4–C26	1.391	1.445
C2–C19	1.399	1.415
C19–C6	1.403	1.385

**Table 2:** Kinetic parameters for the second step of the reaction of Pheo*a* with CuTf_2_ in MeCN in equilibrium with air.

Parameter	k_1_	k_2_
k^298K^	(1.536 ± 0.318) × 10^3^ M^−1^s^−1^	5.309 ± 1.049 s^−1^
ΔH^‡^ (kJmol^−1^)	58 ± 4	71 ± 2
ΔS^‡^ (Jmol^−1^K^−1^)	+10 ± 12	+7 ± 6

## Data Availability

All data are contained within the article.
